# Short-Term Effects of Alfalfa Silage Versus Hay, with or Without Hydrolysable Tannins, on Production Performance, Rumen Fermentation, and Nutrient Digestibility in Mid-Lactation Dairy Cows

**DOI:** 10.3390/microorganisms13102327

**Published:** 2025-10-09

**Authors:** Xuning Miao, Chun Bai, Guofang Chen, Jiajin Sun, He Du, Chen Shen, Wenjie Huo, Qiang Liu, Cong Wang, Lei Chen, Gang Guo

**Affiliations:** 1 College of Animal Science, Shanxi Agricultural University, Jinzhong 030801, China; 13485335899@163.com (X.M.); 18234876101@163.com (C.B.); chengf0036@163.com (G.C.); ss20000729@163.com (J.S.); 15642147811@163.com (H.D.); huohuo-1982@163.com (W.H.); liuqiangabc@163.com (Q.L.); wangdx0321@163.com (C.W.); cl1016zj@126.com (L.C.); 2 Shanxi Provincial School of Animal Husbandry and Veterinary Medicine, Taiyuan 030024, China; shenchen63029@163.com

**Keywords:** dairy cattle, alfalfa, hydrolyzable tannins, rumen fermentation, production performance

## Abstract

This study examined the effects of alfalfa silage versus alfalfa hay in a total mixed ration (TMR) on milk yield, rumen fermentation, and nutrient digestibility in dairy cows. Hydrolyzed tannins (HT) were supplemented individually to assess changes. Thirty-two multiparous Holstein cows (DIM: 94 ± 8 d; milk yield: 41 ± 2 kg) were assigned to four treatments in a 2 × 2 factorial design: basal diet (alfalfa hay, H, or alfalfa silage, S) and additive (control, C, or 100 g/d HT, T). Production performance, rumen fermentation, nutrient digestibility, and blood metabolites were evaluated. Compared with group H, group S had a 0.16% higher milk protein percentage and significantly higher fat-corrected milk yield, milk fat percentage, fat-to-protein ratio, total solids, and milk urea nitrogen. After feeding, the ST group had increased ruminal pH. HT supplementation significantly decreased ruminal NH_3_-N levels (*p* < 0.05) and increased microbial crude protein (MCP) content (*p* < 0.05). Group H showed no significant changes, and the effects of HT were less evident in hay-fed cows than in silage-fed cows. In summary, alfalfa silage feeding increased ruminal microbial populations, while HT supplementation mitigated the post-feeding decline in ruminal pH. Considering the relatively small sample size (*n* = 32), the results should be viewed as indicative rather than conclusive, and future studies with larger cohorts will be valuable to confirm and extend these findings.

## 1. Introduction

Alfalfa is widely used as animal forage due to its high content of protein, vitamins, minerals, and other nutrients [1,2,3]. Known for its drought and cold resistance, alfalfa has a broad growing range and high yield [4]. In livestock production, it is often fed to high-producing ruminants (such as dairy cows) in the form of hay or silage. Alfalfa in hay form provides animals with greater satiety, and its high fiber content promotes intestinal motility; however, the drying process increases the risk of protein loss [5,6]. When ensiled at 30–40% dry matter, alfalfa undergoes anaerobic fermentation, which better preserves its nutritional components and generally provides higher energy and digestible protein compared to hay [7]. Incorporating alfalfa into total mixed rations (TMR) can enhance dry matter intake (DMI), as its palatability and digestibility help improve milk yield, as well as protein and fat content [8]. Therefore, it is important to investigate the effects of different forms of alfalfa on digestion and utilization in high-producing dairy cows.

Processing into hay or silage partially modifies alfalfa protein structure, improving palatability and energy use, but digestive degradation remains limiting [9]. Compared with hay, silage typically shows higher crude protein (CP), neutral detergent fiber (NDF), and acid detergent fiber (ADF), with nearly twice the CP degraded into amino acids, peptides, and NH_3_-N. This raises rumen degradable protein and water-soluble nitrogen, further enhancing digestibility [10]. Rumen microorganisms—bacteria, protozoa, and fungi—are central to energy and protein metabolism [11]. They degrade cellulose and hemicellulose to produce volatile fatty acids (VFAs), mainly acetate and propionate, which supply 70–80% of the cow’s energy. Roughage diets promote cellulolytic microbes, increasing acetate production, a key precursor for milk fat [12]. Propionate, with 20% higher energy efficiency, supports gluconeogenesis and lactose synthesis. In protein metabolism, microbes degrade dietary protein to produce microbial crude protein (MCP), which supplies 60–65% of digestible protein, and assimilate non-protein nitrogen into microbial protein. MCP provides a balanced amino acid profile, with lysine and methionine at ~6.8% and 2.5%, respectively, serving as the main absorbable amino acid source in the small intestine [13]. However, in the rumen, particularly the posterior rumen, a portion of plant-based protein is broken down by microbes, releasing ammonia. Some of this ammonia is subsequently converted into urea and excreted in the urine, leading to environmental pollution. The development of additives capable of leveraging these dynamic microbial functions and interaction mechanisms is crucial for effectively reducing pollutant emissions and enhancing animal production efficiency.

In recent years, a wide range of plant extracts rich in secondary metabolites—including terpenoids, neem extracts, saponins, and flavonoids—have been evaluated for their potential to reduce protein waste [14,15]. Among these, tannins, a class of natural polyphenolic compounds found in shrubs, legumes, cereals, and other plants, have attracted significant attention in protein utilization research. Based on structural differences, tannins are primarily categorized into hydrolyzable tannins (HT) and condensed tannins (CT) [16]. HTs, abundant in gallnuts and chestnut wood, hydrolyze to gallic acid and glucose and generally show stronger biological activity [17]. CTs occur as polymers in legumes [18]. Tannins form stable complexes with proteins, reducing ruminal degradation and traditionally considered anti-nutritional [19,20]. However, they exhibit dual benefits in dairy cattle: ① Protein protection: stable tannin–protein complexes at ruminal pH reduce microbial degradation, allowing more protein to reach the small intestine and improving nitrogen metabolism [21]; ② Microbial modulation: HTs inhibit methanogens while stimulating fibrolytic bacteria, enhancing fermentation and feed digestibility [22]. Appropriate HT supplementation has shown benefits, including reduced methane emissions without impairing protein synthesis. For example, adding 0.72% DM black wattle tannin extract reduced methane by 17% [23,24]. Jones and Mangan (1977) demonstrated that tannins bind with proteins to form complexes that remain stable at ruminal pH but can dissociate under the more acidic pH of the abomasum or the alkaline pH of the duodenum [25]. By reducing protein degradation in the rumen and increasing protein flow to the duodenum, tannins thereby decrease nitrogen loss in urine [26]. Supplementing beef cattle diets with 0.25–1.50% chestnut tannins increased average daily gain by 0.297 kg [18,27].

This study compared the effects of alfalfa hay versus alfalfa silage on rumen fermentation, production performance, nutrient digestibility, and blood metabolic profiles in dairy cattle. In parallel, it investigated the regulatory mechanisms of HT supplementation on the rumen environment, aiming to assess their potential as an alternative to antibiotics in feed additives. The results are expected to provide a scientific basis for enhancing feed utilization and improving the economic viability of ruminant production.

## 2. Materials and Methods

### 2.1. Experimental Animals and Management

The experimental pasture is located at Shanxi Wangxiangyuan Animal Husbandry Co., Ltd., situated west of Gao Village (Beige Town, Taiyuan, China) in December 2022. The animal studies were approved by the Animal Welfare and Ethics Committee of Shanxi Agricultural University. The studies were conducted in accordance with the local legislation and institutional requirements (Approval code. SXAU-EAW-2022C. RD.010025174. Approval Date: 25 November 2022). The alfalfa used in this trial was cultivated in the Yanmen region, Xinzhou, China. The HT additive was purchased from Jiurui Biotechnology Co., Ltd., (Zhangjiajie, China). Each cow received 100 g/day of HT additive with uniform distribution into the TMR.

Thirty-two multiparous Holstein dairy cows (parity 2; DIM: 94 ± 8 d, milk yield: 41 ± 2 kg/d, BW 670 ± 25 kg) were selected for the experiment. They were randomly assigned to four treatment groups in a 2 × 2 factorial design, with eight cows per group (*n* = 8 per treatment): Alfalfa Hay Control (HC), Alfalfa Hay with 100 g/d Hydrolyzed Tannin (HT), Alfalfa Silage Control (SC), and Alfalfa Silage with 100 g/d Hydrolyzed Tannin (ST). All cows were fed twice daily in stalls and received the additive in two equal doses. The study consisted of a 10 d preliminary period followed by a 30 d main trial. During the preliminary period, feed intake, rumination, and fecal characteristics were monitored. Subsequently, changes in body weight, nutrient digestibility, milk yield, milk quality, rumen fermentation, microbial community structure, enzyme activity, and blood biochemical parameters were measured.

### 2.2. Experimental Diet

The dry matter of alfalfa silage was approximately 40%, while the dry matter of alfalfa hay was about 90%. The diet was formulated based on the NRC (2021) [28] guidelines, using 2.25 kg of alfalfa silage to replace 1 kg of alfalfa hay, as shown in Table 1.

### 2.3. Sample Collection and Analytical Methods

This study collected and analyzed multiple indicators in dairy cows to evaluate feed utilization, ruminal fermentation, production performance, and blood metabolic profiles.

#### 2.3.1. Production Performances

Body weight was measured twice: once at the end of the pre-test period and once at the end of the main test period. The body ruler estimation method was used, involving a tape measure to determine chest circumference and body slope length. The body weight was calculated using the formula:

body weight = chest circumference^2^ × body slope length × 90.

#### 2.3.2. Feed and Fecal Samples Collection and Analysis

On the last day of the pre-feeding period and on days 10, 20, and 30 of the formal trial, 500 g each of feed and ort samples were collected, dried at 55 °C for 48 h until a constant weight was reached, ground, sieved (1 mm aperture), and stored in sealed containers for chemical composition analysis. The following components of the ground feed samples were then analyzed: dry matter (DM), crude protein (CP, Kjeldahl method) [29], crude fiber (CF) [29], neutral detergent fiber (NDF), acid detergent fiber (ADF), crude ash (Van Soest method) [30], calcium, and phosphorus [31].

Fecal samples were collected via rectal sampling on the morning of day 27, at noon on day 28, and in the afternoon of day 29. For every 100 g of fresh feces per cow, 25 mL of a 10% tartaric acid solution was added, and the samples were then frozen at −20 °C for TMR digestibility analysis. Before analysis, manure samples were dried at 55 °C for 72 h until a constant weight was achieved, then ground and passed through a 1 mm sieve. The chemical composition of the fecal samples was then analyzed following the same procedures as for the feed samples.

#### 2.3.3. Rumen Content Sampling and Parameter Analysis

Rumen contents: On the morning of day 30, approximately 100 mL of rumen fluid was collected before feeding and 3 h post-feeding using an oral-nasal probe equipped with a metal filter and a manual pump (Chengdu Huazhi Kaiwu Technology Co., Ltd., Chengdu, China). Immediately after collection, the pH of each sample was measured using a pH meter (LE438, Mettler-Toledo Instrument Co., Ltd., Shanghai, China). The samples were subsequently stored at −20 °C for further analysis of volatile fatty acids (VFAs), ammonia N (NH_3_-N), microbial crude protein (MCP), enzyme activity and microbial diversity. The concentration of NH_3_-N in the rumen fluid was determined by the phenol-sodium hypochlorite colorimetric method [32]. The VFA content was measured using gas chromatograph (GC122, Shanghai Precision Scientific Instrument Co., Ltd., Shanghai, China) [33]. Enzymatic activity (Carboxymethyl-cellulase, Pectinase, Cellobiase, Xylanase) was then measured via spectrophotometric colorimetry (UV3000, Shanghai Meipuda Instrument Co., Ltd., Shanghai, China) [34]. Protease activity in rumen fluid was determined by the Folin-phenol method. The MCP content in rumen fluid was measured using the Coomassie brilliant blue colorimetric method [35]. DNA was extracted from rumen microorganisms using the CTAB method. Subsequently, primers synthesized by Beijing Genomics Institute Co., Ltd., (Beijing, China) were used according to the instructions provided with the TaKaRa kit (Beijing Baorui Biological Technology Co., Ltd., Beijing, China). Real-time PCR quantification was carried out using an ABI StepOnePlus instrument (Applied Biosystems, Waltham, MA, USA), achieving amplification efficiencies between 90% and 110% and a standard curve with an R^2^ ≥ 0.999 [36].

#### 2.3.4. Milk Production and DHI Monitor

Milk yield was recorded and milk samples were collected at the end of the pre-test period and on days 9, 19, and 29 of the main test period. A 50 mL composite sample was prepared by mixing milk from the morning, noon, and evening in a 4:3:3 ratio. These samples were then transported at low temperature to the Shanxi DHI Testing Center, where milk components were analyzed using the Fossomatic 5000 series instrument developed by FUCHS in Hellerup, Denmark.

#### 2.3.5. Collection of Blood and Analysis of Serum Indexes

Collection of Blood: On the 30th day of the trial, 30 mL of blood was collected from the tail vein before morning feeding. After allowing the samples to stand at room temperature for 1 h, they were centrifuged at 3000× *g* for 15 min. The serum was then collected, cryopreserved, and analyzed for routine blood indices using a kit from the Nanjing Jiancheng Institute of Bioengineering (Nanjing, China). Glucose (GLU), total protein (TP), albumin (ALB), globulin (GLO), milk urea nitrogen (MUN), calcium (CA), and phosphorus (P) were determined using a UV3000 spectrophotometer (Shanghai Meipuda Instrument Co., Ltd., Shanghai, China). Additionally, total cholesterol (TC), triglycerides (TG), nonesterified fatty acids (NEFA), *β*-hydroxybutyrate (*β*-HB), acetoacetate (ACAC), acetyl-CoA (A-CoA), fatty acid synthase (FAS), adipose triglyceride lipase (ATGL), and other indicators were measured using a microplate reader (Bethon Synergy H1) according to the instructions provided by the Shanghai Duma kit (Shanghai, China).

### 2.4. Data Processing and Statistical Analysis

Data were analyzed using two-way analysis of variance (ANOVA) with SAS 9.2, where factor one was the form of alfalfa supply and factor two was the application of additives. When the interaction effect of the two factors was significant (*p* < 0.05), Tukey’s multiple comparison test was used to compare the means of the groups, with significance set at *p* < 0.05.

## 3. Results

### 3.1. Effect of Forage Form and Hydrolysable Tannins on Apparent Nutrient Digestibility

As can be seen from Table 2, when comparing alfalfa silage with alfalfa hay, apparent digestibility was significantly improved. Apparent DM digestibility was higher in the silage group (85.1% for SC vs. 79.4% for HC; *p* < 0.01). Apparent digestibility of CP, NDF, ADF, and Ca was also greater in the silage group than in the hay group (*p* < 0.05). Supplementation with 1.5% hydrolysable tannins did not significantly affect apparent digestibility of DM, OM, CP, CF, NDF, ADF, Calcium, or Phosphorus (*p* > 0.05). Differences in apparent digestibility were driven primarily by forage form, with no significant interaction between forage form and tannin supplementation (*p* > 0.05).

### 3.2. Rumen Microbiota and Fermentation Responses to Forage Form and Tannins

**Figure 1:**
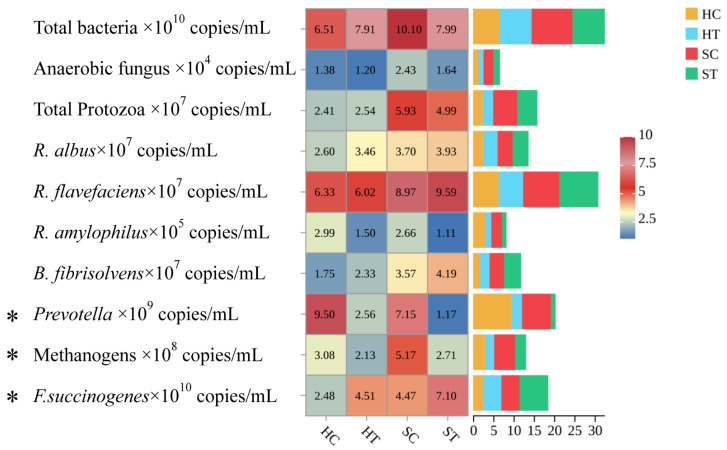
Effect of alfalfa product form and hydrolysed tannins on rumen microbiota structure. * *p* < 0.05. The 2.5–10 indicator diagram indicates the left side of the diagram, with darker colors from bottom to top indicating higher values.

Forage form substantially remodeled the rumen ecosystem. Figure 1 shows that silage-fed cows showed 28% higher fungal counts (*p* < 0.01) and 17% higher *Ruminococcus flavefaciens* abundance (*p* < 0.05); protozoal counts were unaffected by treatments. Tannin-treated groups (both H and S) had significantly lower *Prevotella ruminicola* populations versus controls (*p* < 0.01). It can be observed from Table 3 that together with 5–8% higher carboxymethyl-cellulase and xylanase activities (*p* < 0.01), pectinase activity was lower in S than H (*p* < 0.05). Hydrolysable tannins did not alter carboxymethyl-cellulase, pectinase, cellobiase, or xylanase activities (*p* > 0.05).

Table 4 presents the changes and comparisons of ruminal pH, NH_3_-N, MCP and VFA among the four groups of dairy cows. Rumen fermentation variables also shifted with forage form. MCP concentration was lower in the S group (35.9 mg/dL) compared with H (*p* < 0.05). Isovaleric acid (IVA) decreased after replacing hay with silage (*p* < 0.01), whereas propionic acid (PA), acetate-to-propionate (AA/PA) ratio, and acetate (AA) were higher in S (PA and AA/PA: *p* < 0.01; AA: *p* < 0.05). Tannin inclusion produced modest reductions in ruminal NH_3_-N and methanogen counts (*p* < 0.05) but did not amplify the microbial or digestive advantages associated with silage; these changes were insufficient to improve milk yield or feed efficiency [37].

### 3.3. Effects of Forage Form and Hydrolysable Tannin Supplementation on Intake, Body Weight, and Milk Production

Body weight and production metrics were largely unaffected by forage form or tannin supplementation. As shown in Table 5, the average daily weight gain (DWG) for the HC group was 0.27 kg/d, and for the SC group it was 0.73 kg/d; post-tannin DWG was 0.63 kg/d (HT) and 0.73 kg/d (ST); differences were not significant (*p* > 0.05), indicating no effect on body weight or lactation length. In Table 6, dry matter intake and milk yield were similar between treatments: HC consumed 22.3 kg DM/d and produced 29.7 kg milk/d, while SC consumed 22.6 kg DM/d and produced 29.8 kg milk/d. Supplementing 1.5% hydrolysable tannins (HT, ST) did not change DMI or daily milk yield.

Tannins modestly altered milk composition: milk fat increased from 4.12% to 4.28% and total solids from 12.5% to 12.7% (*p* < 0.05), whereas milk protein remained unchanged. These composition shifts were statistically significant but small and yield-neutral, limiting practical relevance for commercial herds [16].

### 3.4. Effects of Forage Form and Hydrolysable Tannins on Blood Metabolites

According to the data in Table 7, the serum metabolite profiles remained largely stable across treatment groups. Replacing hay with silage did not induce significant changes in serum glucose (GLU), total protein (TP), albumin (ALB), blood urea nitrogen (BUN), calcium (Ca), or phosphorus (P); similarly, no significant differences were observed in total cholesterol (TC), non-esterified fatty acids (NEFA), β-hydroxybutyrate, or fatty acid synthase (FAS). However, the acetoacetate (ACAC) concentration was significantly higher in the S group compared to the H group (*p* < 0.05). Hydrolysable tannin supplementation alone had no significant effect on GLU, serum Ca, or P (*p* > 0.05) but significantly reduced serum TP, globulin (GLO), and BUN (*p* < 0.05). A significant interaction was observed between alfalfa form and tannin supplementation in terms of BUN (*p* < 0.05). Tannins did not significantly affect TC, triglycerides (TG), NEFA, β-hydroxybutyrate, acetyl-CoA, FAS, or adipose triglyceride lipase (ATGL) (*p* > 0.05).

## 4. Discussion

The results of the feeding trials show that neither alfalfa silage nor alfalfa hay directly increases total milk production or growth performance, but it does reprogram nutrient partitioning and metabolic fluxes in the rumen. In this study dairy cows fed alfalfa silage often show no significant change in dry matter intake or overall milk output relative to hay-fed cows, yet milk fat and solids content rise markedly [1]. This suggests that ensiling alters the availability and form of nutrients: silage typically has higher soluble fiber and energy, promoting more extensive ruminal fermentation. As a result, silage diets deliver more digestible energy (especially from fiber) and VFAs per unit intake, without increasing gross intake. Mechanistically, improved fiber digestibility with silage “better stimulates rumen fermentation” and produces more acetogenic precursors for milk fat synthesis [38]. Indeed, silage-fed cows tend to have higher ruminal acetate and lower propionate concentrations, reflecting a shift toward acetate-producing fermentation [39]. This acetate-rich milieu directly fuels de novo fatty acid synthesis in the mammary gland, explaining the elevated milk fat observed with silage diets. In short, forage processing (ensiling) reshapes rumen fermentation: it enhances cellulolytic breakdown by microbes and enriches acetate production, thereby enriching milk’s fat fraction without altering total yield [40].

At the microbial level, silage versus hay alters substrate availability and community structure. Ensiling breaks down plant cell walls and frees sugars, favoring growth of genera such as *Prevotella* that ferment soluble carbohydrates [41]. For example, increased fermentable carbohydrates in silage support *Prevotella* proliferation, which contributes to both amino acid synthesis and SCFA (acetate/propionate) production. Likewise, fiber-degrading phyla like *Fibrobacteres* may proliferate under silage feeding, enhancing cellulolysis (as noted by a 166% increase in *Fibrobacteres* OTUs and a strong correlation between *Fibrobacteres* abundance and milk yield [42].

In contrast, the hay diet with more intact fiber may sustain a higher proportion of *Firmicutes* and *Fiber-degraders* that operate on slower fermentation. Despite these shifts in specific taxa, the dominant phyla (*Bacteroidetes*, *Firmicutes*) remain similar, indicating that overall fiber digestion is maintained across diets [43]. Importantly, beneficial saccharolytic microbes (e.g., *Prevotella*) increase with silage, while several potentially proteolytic or pathogenic genera (e.g., Erysipelatoclostridium, Pseudoflavonifractor, Candidatus Saccharimonas) decline [44]. Together, these changes suggest that silage feeding enhances the rumen’s fermentative capacity (especially fiber breakdown) and yields more substrates (acetate, glucose) for milk synthesis, even though overall digestion kinetics (apparent digestibility of major nutrients) remain largely unchanged [45].

Supplemental hydrolyzable tannins introduce another layer of ruminal modulation. Tannins are polyphenolic plant compounds that bind dietary proteins and microbes. In the rumen, tannins form indigestible complexes with feed proteins, slowing proteolysis [46]. This action “reduces ruminal protein degradability” [47] and lowers ammonia production, because less dietary protein is deaminated by microbes. The net effect is a shift of more undegraded protein to the intestine (enhancing post-ruminal amino acid supply) and improved nitrogen utilization [48]. At the same time, tannins can suppress certain microbial groups (notably methanogenic archaea and proteolytic bacteria), which helps lower methane output and nitrogen losses. By inhibiting protozoa and archaea, tannins decrease hydrogen availability for methanogenesis and shift VFA proportions toward propionate (a more glucogenic VFA) [49].

These combined effects mean that low-dose tannin supplementation in high-alfalfa diets often does not raise milk yield, but it significantly enhances feed efficiency and sustainability. Remarkably, these ruminal changes do not induce major disruptions in systemic metabolism. Blood metabolite profiles are largely stable across treatments, indicating that the animals’ energy and protein homeostasis remain intact [50]. Studies report no significant differences in serum glucose, total protein or general metabolic enzymes when cows are switched from hay to silage [51]. The one exception is blood urea nitrogen (BUN): tannin-fed cows consistently show reduced BUN concentrations. This reduction is mechanistically consistent with less ammonia absorption from the rumen (and hence less hepatic urea synthesis) when protein degradation is curtailed by tannins [52]. A recent in vivo trial found that chestnut tannin extract (hydrolyzable tannins) fed at 0.18–0.36% of diet significantly lowered BUN, confirming the “suppressive effect of chestnut tannins on rumen protein degradation” [53]. Other blood parameters, glucose, nonesterified fatty acids, *β*-hydroxybutyrate, and liver enzymes, remain within normal ranges and show no consistent trends under silage or tannin diets. This indicates that the cows accommodate these feeding strategies without overt metabolic stress. (Interestingly, one study noted slightly higher serum triglycerides with silage, perhaps reflecting increased VFA flux toward lipogenesis for milk fat, but overall lipid homeostasis was preserved.) In sum, the primary action of both forage form and tannin additives is confined to the rumen: they fine-tune fermentation and nitrogen cycling, while systemic energy/protein markers are only minimally affected [54]. Nonetheless, it should be acknowledged that the relatively small sample size in each treatment group (*n* = 8) may limit the statistical power and generalizability of the findings. Future studies involving larger populations and longer feeding periods are warranted to confirm these results and strengthen the evidence base.

## 5. Conclusions

Collectively, the data demonstrate that first, alfalfa silage can fully replace hay without production loss provided fiber digestibility is enhanced, and second, hydrolysable tannins act as targeted modifiers of ruminal nitrogen metabolism rather than broad enhancers of milk yield in high-alfalfa diets. By integrating protein-binding actions with forage matrix effects, hydrolysable tannins unlock nutritional gains while mitigating nitrogen waste, positioning them as dual-purpose tools for environmentally optimized dairy production.

## Figures and Tables

**Table 1:** The composition and nutritional ingredients of the diet.

Item	HC	HT	SC	ST
Ingredients, % of DM				
Corn silage	27	27	27	27
Oaten hay	8	8	8	8
Alfalfa hay	15	15	0	0
Alfalfa silage	0	0	15	15
Concentrate mix ^1^	45	44.7	45	44.7
Additive, % of DM				
Hydrolysable tannin extract ^2^	-	0.3	-	0.3
Nutritive value ^3^				
Net energy for lactating cow (MJ/kg)	6.73	6.73	6.73	6.73
Crude protein (g/kg DM)	178	178	176	176
Neutral detergent fiber (g/kg DM)	514	514	511	511
Acid detergent fiber (g/kg DM)	158	158	157	157
Calcium (g/kg DM)	8.18	8.18	8.22	8.22
Phosphorus (g/kg DM)	5.69	5.69	5.8	5.8

^1^ The concentrate mix consisted of (% of DM): Corn meal (18), Bean pulp (6), Soybean meal (4.5), Spray-dried corn bran (2), Distillers dried grains with solubles (6), Cotton seed (4), Brewers dried grain (5), Stone powder (0.5), Calcium hydrogen phosphate (1), Sodium bicarbonate (1.3), Salt (1), Yeast cultures (0.2), and Premixes of vitamins and minerals (0.5). ^2^ Hydrolysable tannin extract was supplemented at a rate of 100 g/d per cow, which was equivalent to 0.3% of the total DM intake. This amount replaced an equal portion of the concentrate mix in the H + T and S + T diets. ^3^ NEL, net energy for lactating cow; CP, Crude protein; NDF, Neutral detergent fiber; ADF, Acid detergent fiber. The nutritive values shown are for the basal diets (HC and SC). The addition of tannins was not expected to alter these calculated values.

**Table 2:** Effect of alfalfa product form and hydrolysed tannins on the apparent digestibility of nutrients in dairy cows.

Item	Dietary Treatment				SEM	*p*-Value		
	HC ^1^	HT	SC	ST		D ^2^	T	D × T
Dry matter (%)	79.4 ^b^	81.1 ^b^	85.1 ^b^	84.6 ^ab^	0.85	0.0040	0.6682	0.4040
Organic substance (%)	80.9 ^b^	82.2 ^b^	86.2 ^a^	85.7 ^a^	0.80	0.0037	0.7341	0.4540
Crude protein (%)	80.8 ^b^	81.6 ^b^	86.2 ^a^	84.7 ^a^	0.92	0.0217	0.8405	0.4940
Neutral detergent fiber (%)	76.4 ^b^	79.7 ^b^	83.3 ^a^	84.4 ^a^	1.22	0.0155	0.3047	0.6095
Acid detergent fiber (%)	55.7 ^b^	62.4 ^b^	68.5 ^a^	70.4 ^a^	2.46	0.0358	0.3484	0.5920
Crude fat (%)	71.6	75.9	78.8	79.6	1.45	0.0646	0.3516	0.5276
Calcium (%)	56.2 ^b^	58.2 ^b^	67.4 ^a^	66.8 ^a^	2.32	0.0412	0.8765	0.7711
Phosphorus (%)	66.2	67.4	75.7	73.5	1.92	0.0510	0.9077	0.6448

^1^ H, Alfalfa hay group; S, Alfalfa silage group; C, Control group; T, Tannins group. ^2^ D, Alfalfa silage replaces alfalfa hay; T, Additive treatment; D × T, Interaction. ^a,b^ Mean values in the same row with different superscripts differ (*p* < 0.05).

**Table 3:** Effect of alfalfa product form and hydrolysed tannins on Enzyme Activities in dairy cows.

Item	Dietary Treatment				SEM	*p*-Value		
	HC ^2^	HT	SC	ST		D ^3^	T	D × T
Carboxymethyl-cellulase ^1^	0.123 ^b^	0.123 ^b^	0.130 ^a^	0.134 ^a^	0.002	0.0125	0.4686	0.5248
Pectinase	0.593 ^a^	0.587 ^a^	0.533 ^b^	0.533 ^b^	0.008	0.0002	0.7945	0.7819
Cellobiase	0.101	0.103	0.106	0.107	0.001	0.1592	0.4940	0.8476
Xylanase	0.575 ^b^	0.567 ^b^	0.630 ^b^	0.626 ^ab^	0.009	0.0010	0.6983	0.8780

^1^ Activity unit: Carboxymethyl-cellulase (μmol glucose/min/mL); Pectinase (μmol D-galacturonic acid/min/mL); Cellobiase (μmol glucose/min/mL); Xylanase (μmol xylose/min/mL). ^2^ H, Alfalfa hay group; S, Alfalfa silage group; C, Control group; T, Tannins group. ^3^ D, Alfalfa silage replaces alfalfa hay; T, Additive treatment; D × T, Interaction. ^a,b^ Mean values in the same row with different superscripts differ (*p* < 0.05).

**Table 4:** Effect of alfalfa product form and hydrolysed tannins on rumen fermentation parameter.

Item	Dietary Treatment				SEM	*p*-Value		
	HC ^1^	HT	SC	ST		D ^2^	T	D × T
pH	6.51	6.48	6.45	6.46	0.02	0.2620	0.7017	0.6231
NH_3_-N ^3^ (mg/dL)	16.9 ^a^	16.8 ^bc^	15.1 ^b^	12.4 ^ac^	0.52	0.0001	0.0192	0.0259
Microbial protein (mg/dL)	42.0 ^a^	40.5 ^ab^	35.9 ^b^	40.6 ^ab^	0.68	0.0222	0.6226	0.0217
Volatile fatty acids (mmol/L)	119 ^b^	120 ^b^	124 ^a^	124 ^a^	1.46	0.0261	0.7707	0.8548
Acetic acid (mmol/L)	64.7 ^b^	65.2 ^b^	72.7 ^a^	73.0 ^a^	1.35	0.0017	0.8285	0.9740
Propanoic acid (mmol/L)	33.1	33.7	31.1	32.2	0.59	0.1646	0.4894	0.8361
Isobutyric acid (mmol/L)	1.19	1.33	1.31	1.33	0.02	0.1817	0.1030	0.1576
Butyric acid (mmol/L)	15.3 ^a^	15.5 ^a^	14.3 ^b^	13.3 ^b^	0.31	0.0076	0.4483	0.2084
Isovaleric acid (mmol/L)	2.34	2.60	2.51	2.43	0.06	0.9750	0.4531	0.1807
Valeric acid (mmol/L)	1.89	1.55	1.93	1.89	0.11	0.4215	0.4326	0.5394
Propionic acid ratio	1.95 ^b^	1.93 ^b^	2.34 ^a^	2.28 ^a^	0.06	0.0007	0.6611	0.8483

^1^ H, Alfalfa hay group; S, Alfalfa silage group; C, Control group; T, Tannins group. ^2^ D, Alfalfa silage replaces alfalfa hay; T, Additive treatment; D × T, Interaction. ^3^ NH_3_-N, Ammoniacal nitrogen; MCP, Microbial protein; VFA, Volatile fatty acids; AA, Acetic acid; PA, propanoic acid; IBA, Isobutyric acid; BA, butyric acid; IVA, Isovaleric acid; VA, Valeric acid; AA/PA, Propionic acid ratio. ^a–c^ Mean values in the same row with different superscripts differ (*p* < 0.05).

**Table 5:** Lactation period and body weight of dairy cows.

Item	Dietary Treatment				SEM	*p*-Value		
	HC ^1^	HT	SC	ST		D ^2^	T	D × T
Lactation period (d)	100	105	101	104	3.85	0.9623	0.9455	0.9588
Initial weight (kg)	618	617	619	624	7.65	0.5864	0.7011	0.6237
Final weight (kg)	626	636	641	646	8.98	0.3123	0.3621	0.3215

^1^ H, Alfalfa hay group; S, Alfalfa silage group; C, Control group; T, Tannins group. ^2^ D, Alfalfa silage replaces alfalfa hay; T, Additive treatment; D × T, Interaction.

**Table 6:** Effects of alfalfa product forms and hydrolyzed tannins on the performance of dairy cows.

Item	Dietary Treatment				SEM	*p*-Value		
	HC ^1^	HT	SC	ST		D ^2^	T	D × T
Dry matter intake (DMI) (kg/d)	22.3	22.1	22.6	22.7	0.44	0.1563	0.5877	0.5964
Average daily milk yield (kg/d)	29.7	28.6	29.8	30.3	0.33	0.1758	0.6586	0.2207
Fat corrected milk (kg/d)	28.9 ^b^	29.0 ^b^	30.3 ^a^	31.4 ^a^	0.52	0.0007	0.1863	0.3320
Fat (%)	3.85 ^d^	4.05 ^c^	4.38 ^b^	4.50 ^a^	0.07	0.0001	0.0294	0.6144
True protein (%)	3.19 ^b^	3.29 ^b^	3.35 ^a^	3.39 ^a^	0.03	0.0373	0.2279	0.6198
Fat protein ratio	1.21 ^b^	1.24 ^b^	1.31 ^a^	1.33 ^a^	0.02	0.0088	0.4785	0.9301
Lactose (%)	5.02	5.05	4.98	5.04	0.02	0.6624	0.4257	0.7646
Solid no fat (%)	8.21	8.33	8.42	8.23	0.03	0.1077	0.0876	0.9055
Total solids (%)	12.1 ^d^	12.4 ^c^	12.7 ^b^	12.9 ^a^	0.09	0.0001	0.0028	0.5866
Somatic cell count (SCC) (×10^4^ cells/mL)	21.3	22.3	19.5	21.3	1.92	0.8120	0.3127	0.4186
Urea nitroge (mg/dL)	12.8 ^a^	12.5 ^a^	15.8 ^b^	15.2 ^b^	0.51	0.0032	0.5648	0.8144

^1^ H, Alfalfa hay group; S, Alfalfa silage group; C, Control group; T, Tannins group. ^2^ D, Alfalfa silage replaces alfalfa hay; T, Additive treatment; D × T, Interaction. ^a–d^ Mean values in the same row with different superscripts differ (*p* < 0.05).

**Table 7:** Effect of alfalfa product form and hydrolysed tannins on blood indices in dairy cows.

Item	Dietary Treatment				SEM	*p*-Value		
	HC ^1^	HT	SC	ST		D ^2^	T	D × T
Glucose (µmol/L)	337	347	322	409	16.6	0.4669	0.1527	0.2477
Total protein (µg/mL)	882 ^a^	780 ^b^	882 ^a^	783 ^b^	39.1	0.9812	0.0461	0.9802
Albumin (µg/mL)	337	348	329	401	14.6	0.4396	0.0688	0.0603
Globulin (µg/mL)	545 ^a^	432 ^b^	553 ^a^	382 ^b^	45.2	0.8259	0.0471	0.7549
Urea nitrogen (µmol/L)	196	175	225	185	10.5	0.3875	0.0545	0.0622
Blood calcium (mmol/L)	2.37	2.42	2.73	2.57	0.10	0.2176	0.7950	0.6140
Serum phosphorus (mmol/L)	1.22	1.20	1.46	1.29	0.05	0.1215	0.3533	0.4729
Total cholesterol (mmol/L)	13.2	12.9	12.5	11.9	0.24	0.0789	0.3642	0.8176
Triglyceride (mmol/L)	13.1	13.0	13.7	14.0	0.23	0.1006	0.8451	0.6329
Non-esterified fatty acid (µmol/L)	685	681	670	598	16.4	0.1333	0.2429	0.2773
*β*-hydroxybutyric acid (µmol/L)	223	208	213	205	11.6	0.8053	0.6498	0.9035
Acetoacetic acid (µmol/L)	19.3 ^b^	16.7 ^b^	23.3 ^a^	21.5 ^a^	1.10	0.0464	0.2970	0.8310
Acetyl coenzyme A (U/L)	53.2	46.5	40.5	51.5	2.56	0.4567	0.6676	0.1012
Fatty acid synthetase (U/mL)	12.2	10.4	10.6	13.5	0.57	0.4710	0.6015	0.0472
Adipose triglyceride lipase (U/L)	2009	1670	1887	1761	100	0.5442	0.2952	0.6260

^1^ H, Alfalfa hay group; S, Alfalfa silage group; C, Control group; T, Tannins group. ^2^ D, Alfalfa silage replaces alfalfa hay; T, Additive treatment; D × T, Interaction. ^a,b^ Mean values in the same row with different superscripts differ (*p* < 0.05).

## Data Availability

The original contributions presented in this study are included in the article. Further inquiries can be directed to the corresponding author.
